# What The Papers Say

**DOI:** 10.1093/jhps/hnae032

**Published:** 2024-12-10

**Authors:** Ali Bajwa

**Affiliations:** Villar Bajwa Practice, The Princess Grace Hospital, 30 Devonshire St., London W1G 6PU, United Kingdom

## Abstract

The *Journal of Hip Preservation Surgery* (*JHPS*) is not the only place where work in the field of hip preservation can be published. Although our aim is to offer the best of the best, we are continually fascinated by work, which finds its way into journals other than our own. There is much to learn from it, and so *JHPS* has selected six recent and topical subjects for those who seek a summary of what is taking place in our ever-fascinating world of hip preservation. What you see here are the mildly edited abstracts of the original articles, to give them what *JHPS* hopes is a more readable feel. If you are pushed for time, what follows should take you no more than 10 min to read. So here goes…

## THE PREDOMINANT INSERTION OF THE ISCHIOFEMORAL LIGAMENT IS A MERGING TO THE ILIOFEMORAL LIGAMENT AS DEMONSTRATED ON MAGNETIC RESONANCE ARTHROGRAM STUDIES

Hal Martin’s group from Texas, USA [1] states that the capsular ligaments at the hip joint work in synchrony with the acetabulum and femoral head for articular stability. There is a lack of understanding about ischiofemoral ligament (ISFL) anatomy and function. The purpose of their study was to assess the insertion of the ISFL in nonarthritic adult hips.

A retrospective analysis was performed in 72 patients who underwent magnetic resonance arthrogram (MRA) for the assessment of hip pain. The distribution of the ISFL components, the thickness, and the insertion site were assessed by concomitantly using the axial oblique, coronal, and sagittal MRA images. Two insertions of the ISFL anterior to the center of the femoral head were identified in 71 (99%) hips: (i) predominant anterior merging with the iliofemoral ligament as continuation of zona orbicularis, observed in all hips, and (ii) anterolateral junction of femoral neck and greater trochanter. Two ISFL parts (proximal and distal) were identified in 70 (97%) of the 72 studied hips. The proximal part was always thinner (mean 2.6 ± 0.7 mm) and originated from the ischium at the acetabular rim. The distal part was a continuation of the zona orbicularis, and the mean thickness was 6.7 ± 1.6 mm. Both parts merged as they coursed over the superior portion of the femoral head.

The authors concluded that the predominant insertion of the ISFL is a merging to the iliofemoral ligament anteriorly. They felt that surgical procedures such as hip arthroscopy involving the ISFL will affect the function of the iliofemoral ligament, and vice versa.

## HOW IS VARIABILITY IN FEMORAL AND ACETABULAR VERSION ASSOCIATED WITH PRESENTATION AMONG YOUNG ADULTS WITH HIP PAIN?

The authors from Canada [2] state that the acetabular and femoral versions contribute to hip pain in patients with femoroacetabular impingement (FAI) or dysplasia. However, definitions and measurement methods of femoral version have varied in different studies, resulting in different “normal” values being used by clinicians for what should be the same anatomic measurement. This could result in discrepant or even inappropriate treatment recommendations.

The questions they tried to address in this study were that in patients undergoing hip preservation surgery, (i) what is the range of acetabular and femoral versions at presentation, and how much do two commonly used measurement techniques (those of Murphy and Reikerås) differ? (ii) How are differences in acetabular and femoral versions associated with clinical factors and outcome scores at the time of presentation?

This was a retrospective analysis of data gathered in a longitudinally maintained database of patients undergoing hip preservation at a tertiary care referral center. Between June 2020 and December 2021, 282 hips in 258 patients were treated for an isolated labral tear [9% (26 hips)], hip dysplasia [21% (59 hips)], FAI [52% (147 hips)], mixed FAI and dysplasia [17% (47 hips)], or pediatric deformity [slipped capital femoral head epiphysis or Perthes disease; 1% (3 hips)] with hip arthroscopy [71% (200 hips)], periacetabular osteotomy [26% (74 hips)], surgical hip dislocation [2.5% (7 hips)], or femoral derotation osteotomy [0.5% (1 hip)]. They considered those with complete radiographic data (CT including the pelvis and distal femur) and patient-reported outcome scores as potentially eligible. Exclusion criteria were age younger than 18 or older than 55 years (five hips, three patients), signs of hip osteoarthritis (Tönnis grade ≥ 2; 0), pediatric deformity (slipped capital femoral head epiphysis or Perthes disease; three hips, three patients), previous femoral or acetabular osteotomy (two hips, two patients), avascular necrosis of the femoral head (0), history of neuromuscular disorder (Ehlers–Danlos syndrome; three hips, three patients) or rheumatoid disease (ankylosing spondylitis; one hip, one patient), and when CT did not include the knees (19 hips, 19 patients). Based on these criteria, 249 hips in 227 patients were included. Of patients with bilateral symptomatic hips, one side was randomly selected for inclusion, leaving 227 hips in 227 patients for further analysis. The patients’ median age (range) was 34 years (19–55 years), the median BMI (range) was 27 kg/m^2^ (16–55 kg/m^2^), and 63% (144) were female; they were treated with hip arthroscopy [in 74% (168)] or periacetabular osteotomy [in 23% (52)]. Patients underwent a CT scan to measure acetabular and femoral versions using the Murphy (low: <10°; normal: 10°–25°; high: >25°) or Reikerås (low: <5°; normal: 5°–20°; high: >20°) technique. The McKibbin index was calculated (low: <20°; normal: 20°–50°; high: >50°). Based on the central acetabular version and femoral version as measured by Murphy, hips were grouped according to their rotational profile into four groups: unstable rotational profile: high (high acetabular version with high femoral version) or moderate (high acetabular version with normal femoral version or normal acetabular version with high femoral version); normal rotational profile (normal acetabular version with femoral version); compensatory rotational profile (low acetabular version with high femoral version or high acetabular version with low femoral version); and impingement rotational profile (low acetabular version with low femoral version): high (low acetabular version with low femoral version) or moderate (low acetabular version with normal femoral version or normal acetabular version with low femoral version). Radiographic assessments were manually performed on digitized images by two orthopedic residents, and 25% of randomly selected measurements were repeated by the senior author, a fellowship-trained hip preservation and arthroplasty surgeon. Interobserver and intraobserver reliabilities were calculated using the correlation coefficient with a two-way mixed model, showing excellent agreement for Murphy technique measurements {intraclass correlation coefficient 0.908 [95% confidence interval (CI) 0.80–0.97)]} and Reikerås technique measurements [ICC 0.938 (95% CI 0.81–0.97)]. Patient-reported measures were recorded using the International Hip Outcome Tool (iHOT-33) (–100; worse to best).

The mean acetabular version was 18° ± 6°, and the mean femoral version was 24° ± 12° using the Murphy technique and 12° ± 11° with the Reikerås method. Eighty percent (181 of 227) of hips had normal acetabular version, 42% (96 of 227) to 63% (142 to 227) had normal femoral version per Murphy and Reikerås, respectively, and 67% (152 to 227) had a normal McKibbin index. Patients with an impingement profile (low acetabular version or femoral version) were older (39 ± 9 years) than patients with an unstable (high acetabular version or femoral version; 33 ± 9 years), normal (33 ± 9 years), or compensatory (high acetabular version with low femoral version, or vice versa; 33 ± 7 years) rotational profile. Using the Murphy technique, femoral version was 12° greater than with the Reikerås method (*R*^2^ 0.85). There were no differences in iHOT-33 score between different groups (impingement: 32 ± 17 versus normal: 35 ± 21 versus compensated: 34 ± 20 versus unstable: 31 ± 17).

The authors concluded that the variability in femoral version is twice as large as acetabular version. Patients with an impingement rotational profile were older than patients with a normal, compensatory, or unstable profile, indicating that there are other variables not yet fully accounted for that lead to earlier pain and presentation in these groups. Important differences exist between measurement methods. This study shows that different measurement methods for femoral anteversion result in different numbers; if other authors compare their results to those of other studies, they should use equations such as the one suggested in this study, according to the authors.

## THE ROLE OF DIFFERENT ACETABULAR MORPHOLOGIES ON PATIENT-REPORTED OUTCOMES FOLLOWING PERIACETABULAR OSTEOTOMY IN BORDERLINE HIP DYSPLASIA

In this study, Fischer *et al*. [3] discuss the treatment option for borderline hip dysplasia (BHD) that includes hip arthroscopy and periacetabular osteotomy (PAO). To the present day, the controversial discussion remains, which intervention to prefer. Literature reports supporting an educated choice are scarce, based on small patient cohorts, and do not address the variability of acetabular morphology. Consequently, we intended to report PAO outcomes, from patients diagnosed with BHD, dependent on acetabular morphology, in a large patient cohort and aimed to define risk factors for poor clinical results and patient satisfaction.

A prospective monocentre study was conducted. Patients enrolled underwent PAO for symptomatic BHD (Lateral Centre Edge Angle, 18°–25°). A total of 107 hips were included with 94 complete data sets available for evaluation with a minimum follow-up of 1 year and a mean follow-up of 2.3 years. The mean age was 31 ± 8.2 years, and 81.3% were female. As the primary outcome measure, we utilized the modified Harris hip score (mHHS) with minimal clinically important change (MCID) of 8 to define clinical failure. Results were compared after a comprehensive radiographic assessment distinguishing between lateral deficient versus anterior/posterolateral deficient acetabular and stable vs. unstable hip joints.

Overall, clinical success was achieved in 91.5% of patients and the mHHS improved significantly (52 versus. 84.7, *P* < .001). Eight hips failed to achieve the MCID and four had radiographic signs of overcorrection. Comparing variable joint morphologies, the rate of clinical success was higher in patients with an anterior/posterolateral deficient acetabular coverage compared to lateral deficient acetabular (95.2% versus. 90.4%). The highest rate of clinical failure was recorded in unstable hip joints (85.7% versus. 92.5% in stable hips). The authors note that this study demonstrates that PAO is an effective means to treat symptomatic BHD with variable acetabular morphologies, achieving clinical success in 91.5% of all patients. To maintain a high level of safety and patient satisfaction, technical accuracy appears crucial.

## CHATGPT PROVIDES SATISFACTORY BUT OCCASIONALLY INACCURATE ANSWERS TO COMMON PATIENT HIP ARTHROSCOPY QUESTIONS

The authors from Canada and Saudi Arabia [4] assess the ability of ChatGPT to answer common patient questions regarding hip arthroscopy and to analyze the accuracy and appropriateness of its responses.

Ten questions were selected from well-known patient education websites, and ChatGPT (version 3.5) responses to these questions were graded by two fellowship-trained hip preservation surgeons. Responses were analyzed, compared with the current literature, and graded from A to D (A being the highest and D being the lowest) in a grading scale on the basis of the accuracy and completeness of the response. If the grading differed between the two surgeons, a consensus was reached. Inter-rater agreement was calculated. The readability of responses was also assessed using the Flesch–Kincaid Reading Ease Score (FRES) and the Flesch–Kincaid Grade Level (FKGL).

Responses received the following consensus grades: A (50%, *n* = 5), B (30%, *n* = 3), C (10%, *n* = 1), and D (10%, *n* = 1). Inter-rater agreement on the basis of initial individual grading was 30%. The mean FRES was 28.2 (±9.2 SD), corresponding to a college graduate level, ranging from 11.7 to 42.5. The mean FKGL was 14.4 (±1.8 SD), ranging from 12.1 to 18, indicating a college student reading level.

The authors concluded that the ChatGPT can answer common patient questions regarding hip arthroscopy with satisfactory accuracy graded by two high-volume hip arthroscopists; however, incorrect information was identified in more than one instance. Caution must be observed when using ChatGPT for patient education related to hip arthroscopy. Given the increasing number of hip arthroscopies being performed annually, ChatGPT has the potential to aid physicians in educating their patients about this procedure and addressing any questions they may have.

## THE ROLE OF THE HIP CAPSULE IN RESTORING STABILITY IN THE INITIAL PHASE OF HIP DISTRACTION: AN IN VIVO ANALYSIS

The authors from Utah, USA [5] note that the labral suction seal has been shown to provide the majority of resistance in the initial phase of hip distraction. However, the effect of an unrepaired interportal capsulotomy and capsular repair on the initial phase of hip distractive stability *in vivo* is not well understood.

They aimed to investigate the effect of capsular repair on the initial phase of distractive stability of hip joints in patients with femoroacetabular impingement (FAI) syndrome in this laboratory study.

Patients undergoing primary hip arthroscopy for FAI between March and August 2020 were prospectively enrolled. The total joint space was measured on fluoroscopic images at the medial and lateral edges of the sourcil at 12.5-lb (5.7 kg) axial traction intervals [up to 100 lb (45.4 kg)] in three capsular states: (i) native capsule, (ii) interportal capsulotomy, and (iii) capsular repair. Distraction on anteroposterior radiographs was calculated as the difference between the total joint space at each traction interval and the baseline joint space at 0 lb, normalized to millimeters. The native, capsulotomy, and capsular repair states were compared using Wilcoxon signed-rank and McNemar tests. They included 36 hips in 35 patients. The median force required to distract ≥3 mm was 75 lb [34.0 kg; 95% CI, 70–80 lb (31.8–36.3 kg)] in both the native and capsular repair states, which was significantly greater than the median force required to distract ≥3 mm in the capsulotomy state [50 lb (22.7 kg); 95% CI, 45–55 lb (20.4–24.9 kg)]. The most rapid rates of change in joint space were observed at the traction interval at which hips first achieved ≥3 mm of distraction (*n* = 33 hips; 92%).

The traction force at which hips distracted ≥3 mm was 75 lb (34.0 kg) in both the native capsular and capsular repair states. Significantly less traction force (50 lb (22.7 kg)] distracted hips ≥3 mm in the capsulotomy state. Complete capsular closure after interportal capsulotomy resulted in restoration of initial distractive stability relative to the unrepaired capsulotomy state at time zero after primary hip arthroscopy. The authors felt that this study provides surgeons with an improved understanding of the additional stability to the hip joint from capsular repair after hip arthroscopy for FAI syndrome.

## BEYOND THE PATELLA: TREATMENT OF CAM FEMOROACETABULAR IMPINGEMENT SYNDROME IMPROVES ANTERIOR KNEE PAIN

The authors from Spain and Portugal [6] in this study aimed to investigate if there is a relationship between cam femoroacetabular impingement syndrome (cam-FAIS) and chronic anterior knee pain (AKP).

They state that this is a pilot retrospective review of 12 AKP patients with no structural anomalies in the patellofemoral joint and no skeletal malalignment in the lower limbs. All the patients were resistant to proper conservative treatment for AKP (AKP-R). Subsequently, these patients developed pain in the ipsilateral hip several months later and, upon evaluation, were diagnosed with cam-FAIS. Arthroscopic femoral osteoplasty and labral repair were performed and clinical follow-up of hip and knee pain and function [Kujala Score and Non-Arthritic Hip Score (NAHS)] was carried out.

All the patients showed improvement in the knee and hip pain scores with a statistically significant clinical difference in all of them at 69 months of follow-up (range: 18–115) except one patient without improvement in the groin Visual analogical scale (VAS) score postoperatively. The VAS of knee pain improved from 6.3 (range: 5–8) to a postoperative 0.5 (range: 0–3.5). The VAS of groin pain improved from 4.4 (range: 2–8) to a postoperative 0.9 (range: 0–3). NAHS improved from a preoperative 67.9 (range: 28.7–100) to a postoperative 88 (range: 70–100), and knee Kujala’s score improved from a preoperative 48.7 (range: 22–71) to a postoperative 96 (range: 91–100).

They concluded that this study’s principal finding suggests an association between cam-FAIS and AKP-R in young patients who exhibit normal knee imaging and lower limb skeletal alignment. Addressing cam-FAIS in these cases leads to resolution of both groin and knee pain, resulting in improved functional outcomes for both joints.
